# The right to know one’s genetic origins and cross-border medically assisted reproduction

**DOI:** 10.1186/s13584-016-0125-0

**Published:** 2017-01-16

**Authors:** Vardit Ravitsky

**Affiliations:** School of Public Health, University of Montreal, Montreal, Canada

## Abstract

The use of donor sperm or egg for reproduction raises the issue of the right of donor-conceived individuals to know their genetic origins. This paper argues in favor of acknowledging such a right and explores the challenges that cross-border medically assisted reproduction would raise in relation to it. It first explores possible justifications for such a right by discerning its possible conceptual and empirical groundings. It describes some key ethical and policy implications of the removal of donor anonymity. It then argues that novel technologies such as mitochondrial replacement and gene editing raise new concerns in this area and may expand the scope of such a right. Finally, it argues that while many barriers to accessing information about genetic origins already exist at national levels, cross-border medically assisted reproduction may exacerbate a reality in which many individuals conceived through third-party participation are deprived of information that may be crucial to their future well-being for medical or psycho-social reasons.

## Background

In their timely and important article “Ethics and Regulation of Inter-country Medically Assisted Reproduction (MAR): A Call for Action”, which was recently published in the IJHPR, Carmel Shalev and her colleagues call for international, national and professional attention to the governance of cross-border MAR based on a human rights framework, and suggest principles of good practice [30]. This is a thoughtful contribution to a growing bioethical debate surrounding the ethical and policy challenges raised by the global expansion of assisted reproduction and its consequences for the health and well-being of all parties involved.

The article is a report of an inter-disciplinary working group of experts that convened in Israel to discuss these challenges, while dedicating particular attention to the protection of the most vulnerable parties involved in cross-border MAR, i.e. third party service providers and the children conceived through these technologies and interactions. While group members came to a consensus on some key issues related to the way forward in this complex arena, they report disagreement regarding “whether or not children have a right to know the identity of their genetic progenitors”. They agreed that medical professionals have a legal obligation to preserve identifying information about third-party collaborators, but not that those conceived have the right to access this information.

This commentary takes a clear position in relation to this point and argues that children conceived through MAR are entitled to know their genetic origins, i.e. the source of the egg or sperm used in their conception. I will go further and argue that novel technologies such as mitochondrial replacement and even gene editing raise new concerns in this area and may expand the scope of such a right. I will suggest that while many barriers to accessing such information already exist at national levels, cross-border MAR may exacerbate a reality in which many individuals conceived through third-party participation are deprived of information that may be crucial to their future well-being for medical or psycho-social reasons.

## The right to know one’s genetic origins: empirical and conceptual considerations

Individuals conceived with the help of egg or sperm providers are raised by their social-legal parents and may or may not know that another person was involved in their conception. While disclosure of the circumstances of conception is recommended by professional societies [2], birth certificates do not indicate the involvement of a third-party [6, 21]. Disclosure therefore remains the prerogative of parents. Even when parents choose to disclose, access to information regarding the gamete provider may be impossible since anonymous donation is still the norm in most countries and since donor records are often not kept long-term. This clinical and regulatory reality has provoked a heated ethical debate regarding the right of donor-conceived individuals to have access to information about their genetic origins [25].

What arguments underlie the concept of a right to know one’s genetic origins? Two approaches can be found in the bioethics literature [9]. The first is consequentialist and is based on the notion that lack of such knowledge harms donor-conceived individuals and that such harm can be empirically assessed and demonstrated. The second is conceptual and is based on the idea that knowing is a basic human right and as such no empirical support is required to demonstrate what harm occurs when it is violated. I argue based on both approaches that individuals have a right to know their genetic origins and that consequently, clinical or legal frameworks that violate it are ethically unacceptable and should be modified at both national and international levels.

The conceptual approach assumes that people have a right to know their genetic origins regardless of the availability of empirical data to show that lack of knowledge is harmful. To cite Warnock: “I cannot argue that children who are told of their origins (…) are necessarily happier, or better off in any way that can be estimated. But I do believe that if they are not told, they are being wrongly treated” [32]. While not all donor-conceived individuals may indeed suffer harm if their right to access this information is violated, such violation deprives them of the liberty to choose what meaning they assign to the genetic components of their identity and relationships, a choice others in society have. As such, I argue, this significantly limits their autonomy [27].

The empirical approach argues that knowledge of one’s genetic origins is essential for one’s physical and psycho-social well-being and that consequently, lack of access to this information constitutes actual harm. Some focus on the medical aspects of such harm, showing that lack of access poses medical risks and creates health disparities between those who are donor-conceived and those who are raised by genetically-related parents [26, 28]. Medical genetic history is acknowledged as a crucial tool for adopting appropriate preventive strategies, for enhancing diagnostic capacity and for making informed reproductive decisions. Moreover, when parents choose not to disclose to their children that a third-party was involved, these children make false assumptions about half of their genetic history and are thus likely to make uninformed medical decisions.

Others focus on the psycho-social aspects of such harm, arguing that knowledge of genetic origins is important for the development of personal identity and healthy family relationships and therefore violating this right may result in complex identity issues, social challenges and psychological distress, sometimes described as ‘genealogical bewilderment’ [17]. What underlies these notions is the idea that knowing who we are requires knowing how we came to be; that the understanding of oneself requires knowledge of where one’s characteristics and traits came from [19, 20].

It is extremely challenging to empirically assess the claim that lack of access to information about genetic origins leads to significant psycho-social harm [13]. Scant empirical research exists and data are inconclusive [29]. A critical analysis of research evidence conducted in 2012 showed that while most empirical studies have methodological limitations (e.g. selection bias), they “consistently report that most donor-conceived people have an interest in securing information about their genetic and biographical heritage”, concluding that “the evidence is sufficiently robust to promote the implementation of policies and practices that promote transparency and openness in collaborative reproduction, thus reflecting the importance of maximising future choices and opportunities for donor-conceived people” [4].

## Knowing one’s genetic origins: ethical and policy implications

Arguments in favor of allowing donor-conceived individuals access to their genetic origins raise numerous issues. While fully addressing and discussing all of them goes beyond the scope of this paper, this section will briefly describe some of the key issues that are often raised in the debate.

### a/What is meant by a ‘right to know one’s genetic origins’?

The notion of ‘knowing one’s origins’ is complex and can have a variety of meanings for different people. The right to know one’s genetic origins can thus be seen as an umbrella term that covers at least three aspects: the medical aspect, i.e. the right to know one’s full family medical history and to know medically relevant genetic information about the donor; the identity aspect, i.e. the right to personal narrative information about the donor that could assist offspring in completing the picture of their own identity; and the relational aspect, i.e. the right to know the full identity of the donor in order to attempt to establish a relationship with him or her [25].

Each of these aspects of the right involves a different understanding of what it means to be ‘genetically related’ to another person: from a solely biological meaning to a deeper psychological and even existential meaning. Each one also implies different policies. The right to know medical information can be addressed by keeping full and updated donor medical records [26], while the right to attempt a contact or a relationship requires disclosure of full donor identity, as guaranteed for example by UK law (HFEA website [15]).

### b/How does a ‘right to know one’s genetic origins’ impact other parties?

Allowing donor-conceived individuals access to information about their genetic origins obviously has implications for all other stakeholders. It means that the state carries a responsibility to appropriately legislate in the area of family law, to ensure donors do not carry parental liability with regards to any children conceived through their gametes. It means that the state or the industry (e.g. IVF clinics, sperm banks, egg donation agencies) must maintain indefinitely registries or records containing all the relevant information. It means that donors must be fully informed about the implications of open-identity donation and the fact that they are engaging in a potentially life-long commitment to update their records and possibly to meet some day with their genetic offspring. It also means that parents need to be counselled and to consider ahead of time the implications of allowing their children access to such information and the impact this may have on their family unit [18, 24, 25].

### c/Should parents be forced to tell donor-conceived children about the circumstances of their conception?

Securing access to information about genetic origins does not guarantee that donor-conceived individuals indeed have the possibility of accessing it, unless they are aware of the circumstances of their conception [10]. While in same-sex families the issue of gamete donation arises naturally, for heterosexual parents disclosure is a decision they have to make. Many families struggle with this decision, even in jurisdictions that ban anonymous donation. Studies show that the majority of parents choose not to disclose, mentioning as reasons the wish to protect the child from knowledge that is seen as disruptive, to hide the fact of infertility and protect against negative social reactions, and to shield the relationship with the genetically-unrelated parent [7, 12, 16]. This reality raises the complex and multifaceted issue of the right not only to have access to information, but also to know the truth about one’s conception.

One proposed solution to this issue has been to mark birth certificates in a way that would identify those born through gamete donation. This solution, however, is still seen by many as controversial, as it would “compromise the privacy of donor-conceived individuals and/or that of their parents” [6] and would intrude into the intricate fabric of family dynamics. A more acceptable approach is to endorse a culture of openness and acceptance, to enhance educational efforts and to encourage parents to disclose by providing them with counselling and tools [2].

### d/Does a legal ban on anonymous donation reduce the number of donors?

The impact of banning anonymity on the number of those willing to donate gametes is an ongoing concern for those jurisdictions that consider adopting such a policy. Creating an acute shortage of donors is not in the best interest of parents who need third-party assistance in order to conceive. It is also not in the best interest of IVF clinics who wish to appropriately address the needs of their patients. Research regarding the impact of banning anonymity on the availability of gametes is ongoing.

Overall, to date, such research has shown that the number of donors does decrease immediately following a change in policy (i.e. adopting a policy that requires open-identity donation), but also that after a period of time the number of donors can be brought back to the same level by using appropriate recruitment strategies (Adams on Epsify, [1]), [11]. The profile of donors is different in jurisdictions that ban anonymity, but the numbers of donors can be maintained with campaigns that appeal to altruistic motivations. It has been suggested to use an evidence-based approach to the recruitment of gamete donors that takes into consideration the beliefs, attitudes and fears of potential donors [8].

## National and cross-border barriers

While open-identity gamete donation is gradually becoming more prevalent, and while some countries have even banned anonymity [5, 26], anonymous donation is still widespread worldwide and even mandated in some countries, such as Israel [31]. This reality is ethically problematic considering the growing acknowledgement that disclosure to children is recommended [2] and that long-term record keeping is important to allow donor-conceived individuals future access to information [29].

The absence in many countries of supportive legislation and/or local registries to keep information about gamete providers long-term creates barriers to future access to this information. Local norms of practice may also contribute to these barriers, as some clinics may not counsel prospective parents regarding future discussions with their donor-conceived children. Dearth of educational and informational resources in some countries may also create barriers, as parents may lack knowledge regarding the alternatives available to them or regarding how to initiate these types of potentially sensitive conversations with their children.

While such barriers already exist at the national level, cross-border MAR may exacerbate these challenges. For example, a child conceived in one jurisdiction and born in another may have additional barriers to accessing information about her gamete provider. Regulatory frameworks may be different between the two jurisdictions, so that even if she is born in a country that acknowledges the right to access gamete provider information at the age of majority, such as the UK, this information may be kept in a country that does not, such as Israel. Beyond such regulatory barriers, the mere geographic distance may create a barrier to access, since long and costly travel may be required. Finally, language barriers may exist when attempting to find out what information can be obtained, how to do so and in understanding the information if and when it is provided.

At the medical level, screening procedures for gamete providers may differ between countries. Assuming that one’s gamete provider has been screened for certain genetic conditions or other elements of medical history based on local norms, may mislead those conceived with gametes originating from other places. This may create additional medical risks due to false medical assumptions [26].

## Challenges raised by emerging technologies

In their paper, Shalev and colleagues acknowledge that “the issues are here to stay, and will likely grow as new business opportunities emerge to bring to the IMAR market controversial technological innovations, such as the recent developments of mitochondrial replacement therapy, and whole genome sequencing or CRISPR-Cas9 (‘gene editing’) for embryos”. Indeed, in the case of the right to know one’s genetic origins, emerging technologies that may in the future become a part of MAR raise novel challenges. I will discuss two examples of such technologies: mitochondrial replacement and gene editing.

Mitochondrial replacement is a technique that allows women to avoid transmission of mitochondrial disease to their children while still using their own eggs and thus preserving their genetic relatedness to the child. Mitochondria is found in the human cell and produces the energy the cell requires. It contain 37 genes (out of a total of about 30,000) that are currently believed to only play a role related to mitochondrial function and not to the phenotype of the future individual [23]. However, mutations in these genes may result in serious or potentially fatal diseases that currently have no treatment. To prevent the transmission of these detrimental mutations, scientists have proposed to use ‘healthy mitochondria’ from a donor egg to replace that of the intended mother, while still using the mother’s nucleus, which contains the DNA responsible for the genetic identity of the future child. Since the resulting child would have DNA from 3 people (genetic father and 2 women), this technique is often called “3-parent IVF” and has sparked a heated ethical debate [3].

Since children born following mitochondrial replacement would have DNA from 2 women, some have expressed concerns regarding the child’s right to know this technique was used and even the identity of the egg provider, who could be described as a ‘mitochondrial mother’. Arguments in favor of such a right to know highlight the fact that the role mitochondrial DNA (mtDNA) plays in development is still uncertain and that there may be “a precautionary case for granting a right to know: just in case it turns out that mtDNA has a greater role biologically than we thought; or just in case it turns out that MT‐conceived children have a strong desire to know who their donor was” [22]. I argue that in the absence of robust longitudinal data regarding the consequences of mitochondrial replacement, it is in the medical and psycho-social best interest of those conceived to have access to information regarding their mitochondria provider, as in the case of egg provision. However, even in the UK, where anonymous gamete donation is banned, the right to access information about the mitochondrial donor has not been acknowledged.

Mitochondrial replacement is not currently used in IVF clinics and is considered illegal in some countries, such as Canada. In February 2015, the UK became the first country in the world to allow by law clinical trials with this technique. In September 2016, media world-wide reported the birth in April 2015 of the first baby boy conceived through mitochondrial replacement [14]. The IVF was performed for a Jordanian couple by an American Doctor and took place in Mexico, where – to quote the doctor – “there are no rules”. This is a clear case of cross-border MAR performed in a less restrictive jurisdiction to avoid limitations that exist in the home countries of both doctor and patient. This case illustrates how cross-border MAR may complicate and even hinder access to information about genetic origins. This boy’s capacity to access information about his mitochondrial provider would have been limited and unprotected by law even within national boundaries. When doctors and parents travel to another jurisdiction to perform a procedure using a technique that is banned in their home countries, this capacity is significantly reduced.

On a more futuristic note, recent advances in gene editing techniques, in particular CRISPR Cas-9, have sparked an international debate surrounding the ethical implications of germline editing, and the possibility of editing the genome of embryos in a way that would produce individuals whose DNA has been edited before birth [33]. Many ethical arguments have been made, but there is still no literature on the issue of whether a genetically-edited individual would have the right to know that her DNA has been edited, in what way and for what purpose. A full argument in favor of a right to know in this futuristic context is beyond the scope of this commentary, but it is reasonable to argue that if such a right is ever acknowledged, here again cross-border MAR involving gene editing would make it more difficult for those conceived to access this type of information about their genetic origins. This issue also demonstrates that Shalev and her colleagues are right to point to emerging technologies as likely raising new challenges in this area.

## Conclusion

Cross-border MAR indeed raises numerous ethical and policy concerns that need to be urgently addressed. I argue that the right of those conceived through MAR to have access to information regarding their genetic origins is one key element that should inform ethically responsible policies and best-practices that govern cross-border MAR, in order to enhance the protection of the most vulnerable parties. This aspect is of great importance at the level of promoting medical interests, protecting future autonomy and respecting human rights.

## Abbreviations

MAR: Medically Assisted Reproduction
mtDNA: Mitochondrial DNA
